# Renal cell carcinoma metastasizing to duodenum: a rare occurrence

**DOI:** 10.1186/1746-1596-1-29

**Published:** 2006-09-14

**Authors:** Alka Bhatia, Ashim Das, Yashwant Kumar, Rakesh Kochhar

**Affiliations:** 1 Department of Histopathology, Postgraduate Institute of Medical Education and Research, Chandigarh, India; 2 Department of Gastroenterology, Postgraduate Institute of Medical Education and Research, Chandigarh, India

## Abstract

**Background:**

Duodenal metastasis is rare in renal cell carcinoma (RCC) and early detection, especially in case of a solitary mass, helps in planning further therapy.

**Case presentation:**

We present the case report of a 55 year old male with duodenal metastasis of RCC. This patient presented with jaundice and abdominal lump one year after nephrectomy. On upper gastrointestinal endoscopy a submucosal mass lesion was noted in the duodenum, the biopsy of which revealed metastasis.

**Conclusion:**

In a nephrectomized patient presenting with jaundice and an abdominal mass, the possibility of metastasis should be suspected and a complete evaluation, especially endoscopic examination followed by biopsy, should be carried out.

## Background

Renal cell carcinoma (RCC) has a potential to metastasize to almost any site. In descending order of frequency, the most common sites of metastasis are the lung, lymph nodes, liver, bone, adrenal glands, kidney, brain, heart, spleen, intestine, and skin [1]. It can involve any part of the bowel and accounts for 7.1% of all metastatic tumours to small intestine [2]. Duodenal metastasis from RCC is very uncommon and only few cases have been described in the English literature (table 1) [3-18]. Also duodenal metastasis generally occurs when there is widespread nodal and visceral involvement and evidence of metastatic disease elsewhere in the body. Here we present the case report of a patient with duodenal and liver metastasis who presented with jaundice and right sided abdominal lump one year after nephrectomy. Duodenal biopsy performed revealed metastasis in the duodenum.

**Table 1:** Previously reported examples of duodenal metastasis in patients with renal cell carcinoma

**Author**	**Year**	**Age/Sex**	**Duration post-nephrectomy**	**Presenting symptoms**	**Other organs involved**	**Treatment**	**Patient outcome**
Lawson et al [3]	1966	69/F	0 years	Bleeding anemia	-	Pancreatico-duodenectomy	Alive (8 months FU)
Tolia et al [4]	1975	-/M	16 years	-	-	-	5 months
Heymann et al [5]	1978	64/M	8 years	Bleeding	Colon	Complex procedure	3 weeks
McNichols et al [6]	1981	52/M	10 years	Malabsorption	-	Diagnostic only	-
Lynch et al [7]	1987	16/M	1 year	Bleeding	-	Embolization	Alive (6 months FU)
		61/M	6 years	Jaundice	-	Embolization	6 months
		67/M	2 years	Bleeding	Lungs	-	Lost to FU
Robertson et [8]	1990	70/M	13 years	Bleeding	Pancreas	Whipple procedure	-
Gastaca et al [9]	1996	-	8 years	-	-	Duodenectomy	-
Toh et al [10]	1996	59/F	10 years	Obstruction anemia	-	Metastatectomy	Alive (6 months FU)
Ohmura et al [11]	2000	62/M	5 years	Obstruction	-	Embolization- local resection	-
Hashimoto et al [12]	2001	57/M	11 years	Bleeding	Pancreas	PPPD	-
Nabi et al [13]	2001	40/M	4 years	Obstruction	-	Gastrojujenostomy	7 days
Sawh et al [14]	2002	53/M	6 years	Bleeding	Brain Anal canal	Duodenectomy	Alive (4 years FU)
Loualidi et al [15]	2004	76/M	5 years	Anemia	-	Radiotherapy	Alive
Chang et al [16]	2004	63/F	9 years	Bleeding	-	Metastatectomy	-
George et al [16]	2004	65/M	2 years	Obstruction	Omentum ileum	Intestinal Resection	9 months
Arroyo [17]	2005	-	-	-	-	-	-
Bhatia et al (current)	2006	50/M	1 year	Jaundice	Liver	Diagnostic only	Lost to FU

PPPD = Pylorus preserving pancreatico-duodenectomy, FU = Follow up

## Report of a case

**Figure 1:**
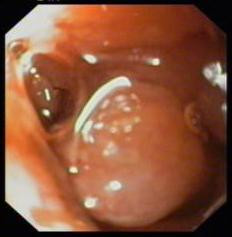
Endoscopic view of submucosal mass lesion in second part of duodenum, with normal glistening mucosa.

The patient was a 55 years old male who came to gastroenterology out patient department with complaints of jaundice and an abdominal mass. He had a history of RCC in the left kidney and had undergone left radical nephrectomy one year ago in our institute. The tumour was present in the lower pole and measured 7 × 5 × 6 cm. Microscopically, it was a conventional clear cell carcinoma (Furhman grade III) involving the renal sinus with tumour emboli in the renal vein. The adrenal gland and ureter were free. This time the patient had jaundice and an abdominal lump. An upper gastrointestinal endoscopy (UGIE) followed by duodenal biopsy was performed. Endoscopy showed a 4 × 4 cm submucosal mass lesion (Fig. 1) in the second part of duodenum. A biopsy was taken from the mass and sent for histopathology.

**Figure 2:**
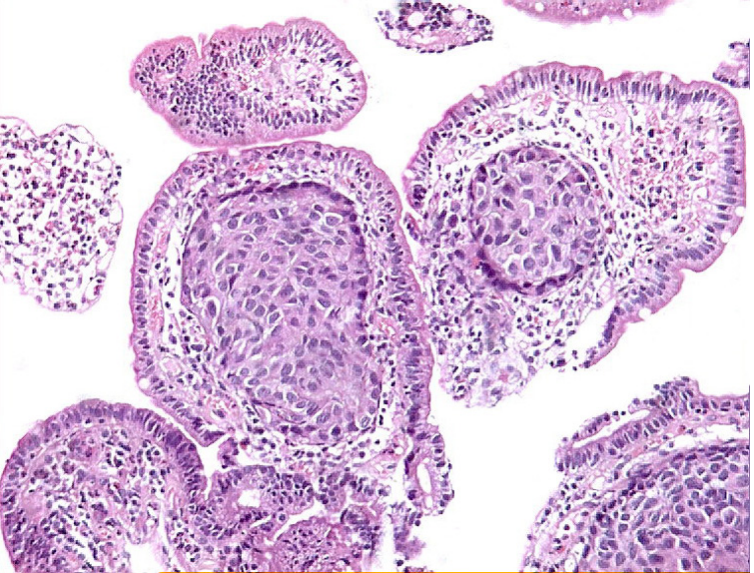
Tumour emboli of renal cell carcinoma in lymphatics of lamina propria.

**Figure 3:**
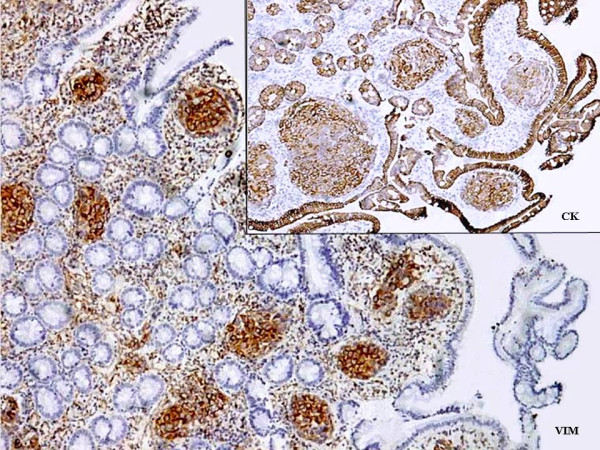
Strong positivity of tumour cells for vimentin and cytokeratin immunostaining.

The biopsy consisted of three fragments which revealed duodenal mucosa with normal villi, however many of the vascular channels in the lamina propria showed tumour emboli. The tumour cells were mildly pleomorphic, had abundant pink cytoplasm and low nuclear:cytoplasmic ratio (Fig. 2). These were positive for cytokeratin (CK), vimentin (Vim) and epithelial membrane antigen immunostaining and negative for chromogranin (Fig. 3). The surrounding lamina propria, villi and crypts were normal. Considering these features, a diagnosis of metastatic renal cell carcinoma was offered on the biopsy. This was followed by abdominal Ultrasonography (USG), CT scan, liver function tests and other investigations to know the extent of illness. Liver enzymes were raised significantly with serum alkaline phosphatase levels of > 1000 IU/L. Both USG and CT scan showed multiple tumour deposits in the liver. Other visceral organs and peritoneum were normal. Radiotherapy as a part of palliative treatment was planned but could not be performed as the patient was lost to follow up.

## Discussion

Small bowel involvement by metastatic tumors is rare and has been reported in only 2% of autopsy cases [2]. Common metastatic malignancies known to involve the small bowel are melanomas, lung cancer, carcinoma of the cervix, RCC, thyroid carcinoma, hepatoma and merkel cell carcinoma. Males are more commonly affected (male: female = 1.5:1) and the incidence of metastasis increases with age [17]. Metastatic lesions of the duodenum are most frequently located in the periampullary region or the duodenal bulb [17]. On endoscopy the lesion can be seen as a submucosal mass with ulceration of the tip, multiple nodules of varying sizes or raised plaques [19]. In the present case the metastatic lesion was seen as a 4 × 4 cm submucosal mass in the 2^nd ^part of duodenum. The patients commonly present with gastrointestinal bleeding or intestinal obstruction [10,17], however our patient presented with jaundice and abdominal lump. On investigation he was found to have liver metastasis also.

The majority of patients are found to have metastasis within a year after nephrectomy though it can be seen even after several years [16]. The routes of spread can be (i) peritoneal dissemination, (ii) direct spread from an intra-abdominal malignancy, (iii) hematogenous and (iv) lymphatic spread [17]. The last two mechanisms can be responsible for metastases in the case reported.

Treatment options in a case of RCC metastasis depend upon the extent and location of the lesion. In the majority of reported cases of duodenal metastasis, metastatectomy was done. However for disseminated malignancy like in our case treatment is in the form of palliative (non-curative) surgery, radiotherapy, chemotherapy (Sunitinib) or immune stimulating agents (Interleukin-2). However even after treatment the patients with metastatic disease have poor survival. The average survival is about 4 months and only 10% of these survive for one year [20].

The report therefore highlights the importance of investigating patients of RCC presenting with any gastro-intestinal tract manifestations for metastasis. A complete evaluation, especially endoscopic examination and biopsy, should be carried out in such patients. Awareness of this entity and a high index of suspicion on the part of the treating physician and pathologist would help in proper diagnosis and treatment.
